# Anti-TNF Therapy in Spondyloarthritis and Related Diseases, Impact on the Immune System and Prediction of Treatment Responses

**DOI:** 10.3389/fimmu.2019.00382

**Published:** 2019-03-19

**Authors:** Silvia Menegatti, Elisabetta Bianchi, Lars Rogge

**Affiliations:** ^1^Immunoregulation Unit, Department of Immunology, Institut Pasteur, Paris, France; ^2^Unité Mixte de Recherche, Institut Pasteur/AP-HP Hôpital Cochin, Paris, France; ^3^Université Paris Diderot, Sorbonne Paris Cité, Paris, France

**Keywords:** spondyloarthritis, anti-TNF therapy, effects of TNF-blockers on the immune system, prediction of responses to anti-TNF therapy, anti-IL-17A therapy, anti-IL-23 therapy

## Abstract

Immune-mediated inflammatory diseases (IMIDs), such as spondyloarthritis (SpA), psoriasis, Crohn's disease (CD), and rheumatoid arthritis (RA) remain challenging illnesses. They often strike at a young age and cause lifelong morbidity, representing a considerable burden for the affected individuals and society. Pioneering studies have revealed the presence of a TNF-dependent proinflammatory cytokine cascade in several IMIDs, and the introduction of anti-TNF therapy 20 years ago has proven effective to reduce inflammation and clinical symptoms in RA, SpA, and other IMID, providing unprecedented clinical benefits and a valid alternative in case of failure or intolerable adverse effects of conventional disease-modifying antirheumatic drugs (DMARDs, for RA) or non-steroidal anti-inflammatory drugs (NSAIDs, for SpA). However, our understanding of how TNF inhibitors (TNFi) affect the immune system in patients is limited. This question is relevant because anti-TNF therapy has been associated with infectious complications. Furthermore, clinical efficacy of TNFi is limited by a high rate of non-responsiveness (30–40%) in RA, SpA, and other IMID, exposing a substantial fraction of patients to side-effects without clinical benefit. Despite the extensive use of TNFi, it is still not possible to determine which patients will respond to TNFi before treatment initiation. The recent introduction of antibodies blocking IL-17 has expanded the therapeutic options for SpA, as well as psoriasis and psoriatic arthritis. It is therefore essential to develop tools to guide treatment decisions for patients affected by SpA and other IMID, both to optimize clinical care and contain health care costs. After a brief overview of the biology of TNF, its receptors and currently used TNFi in the clinics, we summarize the progress that has been made to increase our understanding of the action of TNFi on the immune system in patients. We then summarize efforts dedicated to identify biomarkers that can predict treatment responses to TNFi and we conclude with a section dedicated to the recently introduced inhibitors of IL-17A and IL-23 in SpA and related diseases. The focus of this review is on SpA, however, we also refer to RA on topics for which only limited information is available on SpA in the literature.

## Introduction

### Immune-Mediated Inflammatory Diseases—An Overview

Immune-mediated inflammatory diseases (IMID) is a term used to define a group of clinically heterogeneous, unrelated conditions that share common inflammatory pathways and derive from aberrant immune responses of the human adaptive or innate immune system. Overall, the estimated incidence of IMIDs in Western populations approximates 5–7% (1) and encompasses over 100 different clinical disorders such as rheumatoid arthritis (RA), inflammatory bowel disease (IBD), spondyloarthritis (SpA) or ankylosing spondylitis (AS), systemic lupus erythematosus (SLE), and psoriasis. The immune dysregulation in IMIDs causes significant morbidity and is a considerable burden for the patients in terms of pain, limited mobility and diminished quality of life, as well as for the society, because of the associated high health-care costs, and the loss of productivity. Our understanding of the pathogenic mechanisms involved in these diseases remains very limited but recent advances revealed that they are likely to derive from a complex interplay between extrinsic environmental triggers and genetic risk factors (2). Several environmental factors have been recognized to play an important role in the risk of developing immune-mediated inflammatory diseases, including smoking, diet, excess alcohol, antibiotic intake, infections, and socioeconomic status (3, 4). However, there is limited evidence of their causality with respect to IMIDs.

Recent genome-wide association studies (GWAS) performed with thousands of patients and controls from different populations have provided detailed information about the genetic variants associated with immune-mediated inflammatory diseases (5). These studies have brought to the forefront many genes linked to signaling pathways that were not known to be involved in the pathogenesis, pointing to new directions in the study of disease mechanisms. At present, more than 600 loci affecting susceptibility to chronic inflammation and/or autoimmune disorders have been mapped by GWAS, revealing many loci that are common to several immune-mediated disorders, suggesting that these conditions may share pathways (6–8). A recent meta-analysis of multiple sclerosis (MS) combining independent GWAS results and genotyping of 80,000 cases and controls, revealed 110 non-MHC risk loci, the majority of which were mapped in the proximity of genes involved in different immune processes. Moreover, the genetic risk loci identified for MS have prominent intersections with loci for other chronic inflammatory diseases, such as IBD, ulcerative colitis, Crohn's disease, celiac disease, rheumatoid arthritis and psoriasis (7, 9, 10). Another recently published meta-analysis of IBD identified a total of 163 genetic risk loci, of which one third was found to overlap with loci previously identified in other inflammatory and autoimmune diseases (7, 11). Therefore, GWAS provided fundamental evidence for a key role of the immune system in the pathogenesis of these diseases, as many of the identified loci map to genes involved in different immune processes. However, for most single nucleotide polymorphisms (SNPs), the mechanisms by which they affect pathogenesis and the targeted cell populations are still unknown.

Frequently, multiple immune-mediated inflammatory disorders co-exist within the same patient. This was observed in a large study involving 3,287 AS patients, of which the 39% also developed uveitis, 16% psoriasis, and 8% inflammatory bowel disease (12). In addition, different IMIDs may co-exist within the same family (13). Considerable progress in the classification of these different disorders in the same group of diseases derives from the introduction in the clinic of tumor necrosis factor-α (TNFα) inhibitors, demonstrating clinical benefit in a number of different diseases, such as rheumatoid arthritis, Crohn's disease, psoriasis, and AS and this concept has been used to establish a cytokine-based disease taxonomy (7, 14). Taken together, this information provides evidence that immune-mediated inflammatory diseases are complex disorders that may share pathogenic mechanisms and triggers, such as environmental factors and genetic susceptibility, so that different diseases may be present in the same patient. Our limited understanding of the pathogenic mechanisms involved in these diseases currently hinders early diagnosis and the development of more specific and effective therapies.

### Biology of TNF-α

Cloned and characterized by Pennica et al. (15), Tumor Necrosis Factor alpha (TNF-α) is a potent pro-inflammatory cytokine secreted by different immune cells, such as activated NK and T-cells, macrophages, monocytes, and neutrophils. TNF-α is also produced by non-immune cells, including fibroblasts and endothelial cells (16). Monocytes and macrophages are the primary source of TNF-α in response to inflammatory stimuli (17). At the transcriptional level, *TNF* mRNA is induced by the cooperation of AP-1 transcription factors with nuclear factor associated with activated T cells (NFAT) and nuclear factor-kB (NF-kB), which can bind directly to the promoter of the *TNF* gene (18).

The first studies on TNF-α characterized its biological function as a potent tumoricidal, in particular as an inducer of tumor hemorrhagic necrosis *in vivo* and a promoter of programmed cell death (apoptosis) *in vitro* (19). Subsequent studies have shown that TNF-α is implicated in a wide spectrum of biological effects. In the immune system, these include: (i) promoting monocyte/macrophage differentiation (20, 21); (ii) enhancing activated B cell proliferation (22, 23); (iii) inducing inflammation, often acting together with IL-1β (24), to protect against viral and bacterial infections (i.e., Mycobacteria tuberculosis) (25). Other functions of TNF include mediation of cachexia, apoptosis, regulation of cell proliferation and maturation of myeloid cells [reviewed by (26)].

The pleiotropic effects of TNF can be understood by the complexity of the signaling pathways activated. Like most of the TNF super family members, TNF-α is synthesized as a 26 kDa type II transmembrane protein (tmTNF), which is subsequently cleaved in the extracellular domain by the metalloprotease TNF-converting enzyme (TACE, also called ADAM-17), resulting in the release of the mature soluble TNF monomer (sTNF), a protein of 17 kDa (27, 28). The same enzyme can cleave TNF receptors (TNFRs) from the cell surface, a mechanism that downregulates receptor expression and releases circulating TNFRs that may act as inhibitors. Both soluble and mTNF can be found as monomers, or assembled in biologically active trimers of 51 kDa.

**Figure 1:**
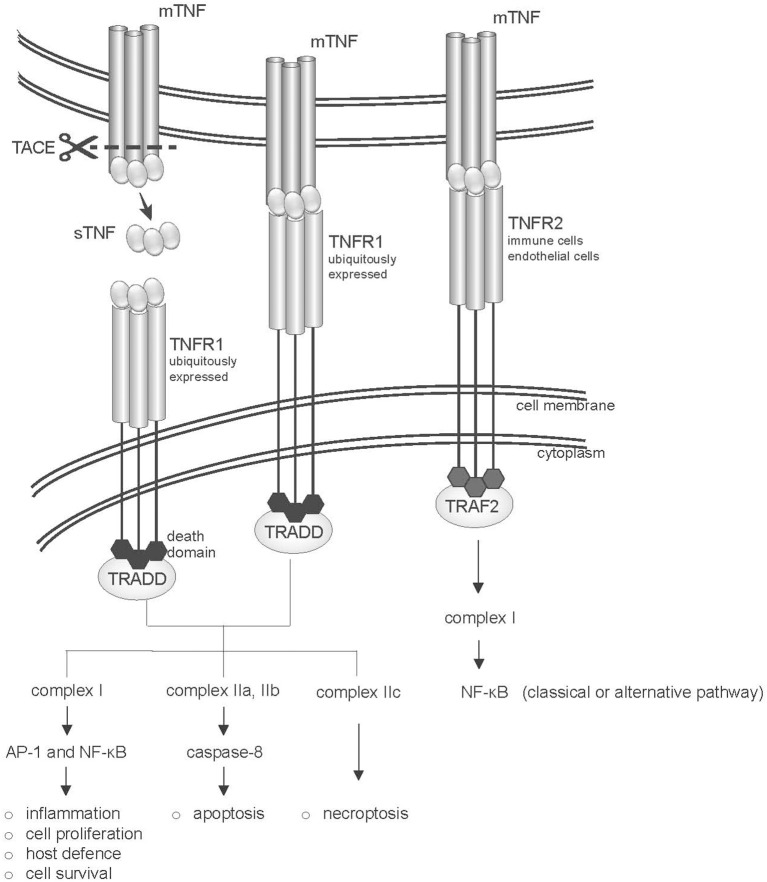
Structure of the TNF-TNFR system. The two TNF receptors (TNFR1 and TNFR2) are shown. TNFR1 and TNFR2 bind both soluble (sTNF) and transmembrane-TNF (mTNF) trimers, however TNFR2 is mainly activated by mTNF. TNFR1 is ubiquitously expressed and in its intracellular portion bears a “death domain” motif (dd), which recruits the adaptor protein TNFR1-associated death domain protein (TRADD). Binding of TNF to TNFR1 leads to the activation of several pathways, including inflammation, tissue degeneration, cell survival and proliferation or alternatively apoptosis or necroptosis. TNFR2 recruits TNFR-associated factor 2 (TRAF2) via its TRAF domain, activating the classical or alternative NF-kB pathways.

TNF-α exerts its activity by binding to two different receptors that differ in cellular localization and signaling mechanisms (Figure 1). Like their ligands, both TNFR1 and TNFR2 receptors are trimerized in biological active complexes through a conserved domain in their extracellular region that mediates ligand-independent receptor assembly. While TNFR1 is engaged by both soluble and membrane-bound TNF, TNFR2 is thought to be mainly activated by mTNF (29, 30). However, both receptors are co-expressed on immune cell types, and it has been suggested that they could also signal cooperatively (31, 32).

The 55-kDa TNFR1 (also known as p55 or CD120a, encoded by *TNFRSF1A*) is ubiquitously expressed (except for erythrocytes), and is characterized by the presence in its intracellular portion of a “death domain” motif. Binding of TNF-α to TNFR1 initiates a complex network of downstream events that may result in both the induction of apoptosis and of acute inflammation. Upon activation, TNFR1 recruits TNFR1-associated death domain protein (TRADD) to the plasma membrane, followed by the assembly of a scaffolding signaling complex (complex I) that includes TNF receptor associated factor 2 (TRAF2), and that results in the activation of AP-1 and NF-kB transcription factors (33, 34). The activation of these transcriptional pathways leads to the expression of genes involved in the defense against pathogens, inflammation, cell proliferation and survival (see https://www.bu.edu/nf-kb/gene-resources/target-genes/) (35). Among the genes induced by NF-kB are many chemokines and cytokines (including IL-6, IL-1β, and IFNγ), as well as several anti-apoptotic factors, such as cIAP-1, cIAP-2, cFLIP, TRAF1, and TRAF2 (36), that suppress caspase 8 activation. On the other hand, TNF binding also induces internalization of TNFR1 in the endocytic compartment, which is crucial for the recruitment to the receptor of TRADD-FADD containing complexes (complexes IIa, b, and c). Complexes IIa and IIb promote cleavage of caspase 8 and apoptosis (35, 37, 38). Complexe IIc activates the process of necroptosis, that is, programmed cell death associated with rupture of the plasma membrane and release of molecules that elicit inflammation (39).

The relevance of TNFR1 for immune-mediate diseases is supported by the association of genetic variations at the *TNFRSF1A* locus with AS, primary biliary cirrhosis and MS (https://www.immunobase.org/). A genetic variant identified selectively in multiple sclerosis has been demonstrated to affect splicing and induce expression of a soluble form of the receptor, which can block TNF. Of note, this variant has not been identified in diseases that are ameliorated by inhibition of TNF, such RA, psoriasis, and Crohn's disease (40).

The 75-kDa TNFR2 (also known as p75/p80 or CD120b, encoded by *TNFRSF1B*) is mainly expressed on lymphocytes, endothelial cells and astrocytes or oligodendrocytes (33), and undergoes transcriptional and posttranscriptional regulation in response to external stimuli (41). TNFR2 preferentially binds mTNF (29), and lacks a death domain. The intracellular portion of TNFR2 presents a transmembrane immunoglobulin and mucin (TIM) domain, which interacts with TRAF adaptor proteins (33, 34). Signaling occurs through the assembly of a complex I containing as a major adaptor protein TRAF2. This leads to the activation of the JNK kinase and of the AP1 transcriptional complex, and of NF-κB signaling through both the classical and alternative pathways (42, 43). Signaling through TNFR2 is subject to an autoregulatory loop, where TRAF2 undergoes ubiquitin-dependent degradation, following TNFR2 activation (44).

A biallelic polymorphism in exon 6 of TNFR2 has been described to result in a non-conservative amino acid substitution (methionine to arginine at codon 196) in the membrane proximal region of TNFR2 (45). This variant has been associated with chronic inflammatory disorders, such as systemic lupus erythematosus, familial rheumatoid arthritis, and ulcerative colitis (46, 47). The mutated receptor shows reduced recruitment of TRAF2 upon TNF-α stimulation and reduced NF-kB activation (48).

The important pathogenetic role of TNF-α in chronic inflammatory diseases is supported by the therapeutic efficacy of anti-TNF agents.

### The Advent of TNF Inhibitors

As mentioned above, TNF-α was initially considered as a potential onco-therapeutic agent. However, despite the name, administration of recombinant TNF-α to patients with malignant diseases resulted in disease progression and severe side effects, rather than disease improvement (49). Phase I clinical trials showed dose-dependent acute toxicities including fevers, chills, nausea, and confusion. Once its role as an early and primary cytokine implicated in the inflammatory immune response had been established, several studies on animal models demonstrated the central importance of TNF-α in the pathogenesis of a wide range of immune-mediated inflammatory diseases, suggesting its blockade as a therapeutic approach. Beutler and colleagues showed that neutralizing antibodies against TNF protected the animals against TNF-mediated endotoxemia (50). The idea of using compounds blocking TNF-α was further supported by the increased levels of TNF-α in the serum or tissues of patients with inflammatory diseases or infections, and in individuals affected by sepsis (51, 52). The concept that blocking a single pro-inflammatory cytokine such as TNF-α could restore homeostasis of a complex network and ameliorate signs and symptoms of chronic inflammatory diseases was a real breakthrough in medical practice. Originally developed for the treatment of rheumatoid arthritis (RA), the therapeutic employment of anti-TNF agents was extended to the treatment of ankylosing spondylitis in the early 2000 (53–55).

Initially, treatment options for IMID were limited to non-steroidal anti-inflammatory drugs (NSAIDs) and physical therapy. At present, NSAIDs such as ibuprofen, naproxen, diclofenac, celecoxib, highly effective in reducing stiffness and back pain in axial SpA, are recommended by the ASAS/EULAR guidelines as first-line treatment (56). When patients do not respond to, or do not tolerate NSAIDs, anti-TNF agents are approved as a second step of medical intervention (57). TNF-neutralization has been very successful for the treatment of SpA in the past decade, however, as for other chronic inflammatory diseases, 30–40% of SpA patients do not respond or respond inadequately to the therapy. In clinical practice, non-responsive patients are treated with various molecules until an effective therapeutic agent is identified. However, this procedure is expensive and may take a long time, during which the patient is not appropriately treated and is exposed to side effects without clinical benefit.

The most common side effect observed during anti-TNF therapy is the increased risk for serious chronic infections, in particular affecting the respiratory tract. Reactivation of latent tuberculosis (TB) remains indeed the major concern (58). Other infections are also recurrent, such as histoplasmosis, Pneumocystis *pneumonia*, influenza, and adenovirus infections, latent viral infections (varicella-zoster, herpes-zoster), skin and soft tissue infections and urinary tract infections. Cases of exacerbated legionella were also reported. More rare side effects include severe hepatic reactions, nervous system disorders, hypersensitivity reactions and leucopenia. Some meta-analyses have been performed to assess the increased risk of malignancies following anti-TNF therapy and there is no proven increase of any malignancy so far. An increased risk of non-melanoma skin cancer during therapy with TNF-blockers has been reported (59, 60). Increased frequencies of anti-double-stranded DNA antibodies have been observed in some patients (61).

### TNF Inhibitors Currently in Clinical Use

**Figure 2:**
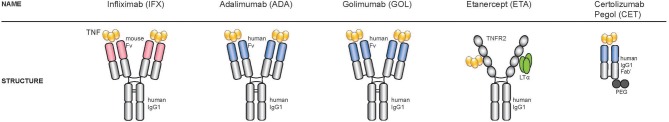
Structure of the five TNF-inhibitors approved for the treatment of spondyloarthritis. Starting from the left: three TNF-inhibitors are full-length bivalent IgG monoclonal antibodies (infliximab, adalimumab, golimumab), one is a soluble receptor (etanercept) and one a PEGylated Fab fragment of a monoclonal antibody (certolizumab).

Natural and engineered anti-TNF antibodies share a similar structure (Figure 2), constituted of 2 heavy (H) chains and 2 light (L) chains connected by disulphide bonds at the hinge region. The two chains contains a variable region (V) in the N-terminal domains (V_H_ and V_L_), that recognizes the target and a C-terminal constant region (Fc). The engineered monoclonal antibody monomer comprises a variable region that can be either murine or human, and a constant region (Fc), usually human to preserve favorable pharmacokinetic properties.

At present, there are five TNF-inhibitors approved for the treatment of spondyloarthritis in the UE, the USA and many other countries (57). Three are full-length bivalent IgG monoclonal antibodies (adalimumab, golimumab, infliximab), one is a soluble receptor (etanercept) and one a PEGylated Fab fragment of a monoclonal antibody (certolizumab).

The first TNF blocker introduced in the clinic for the treatment of RA was the monoclonal antibody infliximab (trade name Remicade) (62). Infliximab is a chimeric human IgG1 antibody linked to murine immunoglobulin variable regions with specificity for human TNF (63, 64). Produced by hybridoma cells, infliximab can bind both soluble and membrane-bound TNF-α, therefore preventing the interaction of TNF with its receptors. Infliximab is administered intravenously and as a consequence requires “day hospital” treatment.

Adalimumab (commercial name Humira) and golimumab (Simponi) (65), are fully human monoclonal antibodies. Adalimumab was developed using phage display, golimumab is obtained from the immunization with TNF of transgenic mice expressing human IgGs (66) and both antibodies are potentially less immunogenic than infliximab.

A different type of anti-TNF compound is etanercept (commercial name Enbrel), an engineered dimer composed of 2 extra-cellular portions of the human p75 TNF-α receptor (TNFR2) fused to a C-terminal human IgG1 Fc domain (67). Etanercept was the first recombinant receptor:Ig fusion protein to be approved for therapeutic use (26). Like adalimumab and golimumab, etanercept is administered subcutaneously. It is produced in Chinese hamster ovary (CHO) mammalian cells, has increased affinity for soluble TNF-α and decreased serum half-life, compared to the monoclonal antibodies (68–71). Whether etanercept also binds to transmembrane TNF-α (mTNF) is still a matter of debate. Some groups showed that the binding affinities/avidities of monoclonal antibodies and etanercept to mTNF were similar (68, 71, 72), whereas other studies reported that infliximab and adalimumab bind to mTNF-α with 3-fold greater avidity than etanercept or certolizumab. However, a more recent study from Kaymakcalan and colleagues, demonstrated that infliximab, adalimumab, and etanercept bind sTNF and mTNF with similar characteristics (69) in transfected cell lines and primary peripheral blood mononuclear cells (PBMCs) isolated from healthy donors, highlighting the ability of etanercept to also interact with the transmembrane form of TNF-α. In addition to TNF-α, etanercept binds to lymphotoxin (LT-α or TNFSF1), a related member of the TNF family that also binds to TNF receptors (TNFR1 and TNFR2) (16). Whether the concomitant blockade of lymphotoxin by etanercept improves clinical responses in patients has still to be fully elucidated. In a large cohort of RA patients enrolled in the CORRONA study, the authors observed that drug response or remission outcomes were similar for patients treated with etanercept and patients treated with anti-TNF-α antibodies (adalimumab, infliximab), except for the fact that response, remission and persistence rates were lower for patients who switched anti-TNF (73). In addition, RA patients treated with pateclizumab, an anti-lymphotoxin-α antibody, did not show statistically significant therapeutic responses as compared to placebo. Response rates were much lower than in RA patients treated with adalimumab in the same clinical study, suggesting that TNF-α blockade has a prevalent role in the improvement of signs and symptoms of RA over LT-α blockade (74). One clinical case involving only one RA patient, reports that the primary non-responder patient initially treated with infliximab, obtained a better response after switching to etanercept. The authors discussed that the responsiveness to etanercept was due to the presence of high levels of LT-α in the synovial tissue as assessed in a biopsy specimen and that resistance to TNF blockade might happen when TNF-α is not the dominant inflammatory cytokine (75). Interestingly, while infliximab and adalimumab have demonstrated efficacy for Crohn's disease (76), etanercept has not shown therapeutic benefits in this disease (77). Differences in agent design and affinity to TNF-α could also explain the differences in infection rates between patients treated with etanercept or with monoclonal antibodies (78–80). There is no evidence that the concomitant blockade of TNF-α and LT-α by etanercept increases mycobacterial infection rates. In a French study including patients with different IMID and treated with TNFi, the risk of tuberculosis was rather higher for those receiving monoclonal antibodies than the soluble-receptor etanercept (80). The differences in design and affinity might perhaps also explain why some patients do not respond to one type of TNF blocker but can achieve significant clinical response by switching to a different type (81).

Certolizumab pegol is the most recent anti-TNF compound introduced in the clinic. Certolizumab is a humanized anti-TNF monoclonal antibody that contains murine and human amino-acid sequences within the V_H_ and V_L_ domains. The hinge region of certolizumab is modified by polyethylene glycol, which reduces immunogenicity, improves solubility and the *in vivo* half-life (68).

When anti-TNF antibodies bind to tmTNF, they may also induce Fc-mediated effects, such as antibody-dependent cellular cytotoxicity (ADCC) or complement-dependent (CDC), although these effects have never been demonstrated in patients (68, 72, 82). All anti-TNF agents, except certolizumab (68), can induce ADCC, whereas etanercept, in contrast to monoclonal antibodies, lacks CDC activities (72). In a process called “reverse signaling” TNF inhibitors may also trigger in tmTNF-positive cells diverse intracellular signals, that inhibit cell proliferation, induce apoptosis, affect inflammatory cytokines and chemokines production, or, conversely, promote cell activation [reviewed by (83)].

Given the complexity of the cellular pathways involved, the molecular mechanism of action of anti-TNF agents is still not fully elucidated. In general, the biological effects of anti-TNF agents can be summarized by these main functions: (i) binding of soluble TNF-α and LT-α, reducing the signaling cascades downstream of these cytokines; (ii) binding to FcR-expressing cells, inducing antibody-mediated cellular cytotoxicity; (iii) binding to membrane-bound TNF (tmTNF), triggering reverse signaling.

## Effects of anti-TNF therapy on the immune system

Given the pleiotropic functions of TNFα, its blockade has long ranging effects on a variety of immune cells. Tables 1, 2 summarize a few of the many studies conducted in rheumatic diseases on the effect of anti-TNF treatment, with a focus on changes in cell populations in Table 1 and secreted cytokines in Table 2. A large number of these studies have analyzed T cell function, since it has been largely demonstrated that these cells are affected by exposure to TNF (158, 159). In experimental models and in humans, long-term exposure to TNFα induced downregulation of components of the T cell receptor (TCR) complex, with consequent reduced responses to TCR-mediated stimulation (160, 161). TNF-blockade was found to reverse the functional impairment, and promote T cell proliferation (160), explaining the increase in memory (91, 127) or effector T cell populations observed in patients treated with TNF inhibitors (see Tables 1, 2) (144).

**Table 1:** Immune cell subsets and anti-TNF treatment.

**Pathology**		**Treatment**	**Subjects**	**References**
**T HELPER CELLS**				
RA	**Flow cytometry** Peripheral blood: increased Th17 vs. controls Synovial fluid: increased Th1, Th17, IL-17+IFNγ+ CD4, TNFα+ CD4		38 RA	Gullick (84)
RA	**Flow cytometry of intracellular and secreted cytokines from PBMC** Increased frequency of CD4+IL-17+ cells and CD4+INFγ+ cells in RA patients in remission, compared to active RA or to controls. FNγ production lower in patients with active disease, compared to controls. No significant changes in Th17 cells in all pooled RA, compared to controls.	ADA	54 HC 243 RA	Aerts et al. (85)
RA	**Flow cytometry, ELISA** Higher frequencies of circulating Th17 cells in active RA patients than in healthy controls. High baseline level of IL-17 is associated with poor therapeutic response.	ETA, ADA	12 HC 48 RA	Chen et al. (86)
RA	**Flow cytometry and Optical LiveCell Array** anti-TNF treated: decreased total CXCR3+, CD4+ CXCR3+, CD4+IL-12R+ cells; increased CD4+CCR4+, CD4+IL-4R+ cells	ETA, ADA, IFX	9 HC 46 RA	Herman et al. (87)
RA	**Flow cytometry, ELISA** RA vs. healthy controls: increased Th1, Th17; decreased Th2, Treg After therapy: decreased Th1, Th17; increased Treg	ETA	10 HC 40 RA	Lina et al. (88)
RAAS	**Flow cytometry, Luminex** Increased Th1, Th17 and Treg in AS, compared to healthy controls (unstimulated PBMC). Decreased after anti-TNF treatment. Increased Th1 in RA, compared to healthy controls.	ETA, IFX	25 HC 20 RA 46 AS	Limon-Camacho et al. (89)
RA	**Flow cytometry, ELISA** Increase in circulating Th17 cells after anti-TNFα therapy. Increased production of IL-12/23p40 in stimulated blood, PBMC or monocytes after anti-TNF therapy, correlated to non-response. PBMC from non-responder have increased IL-17 production upon stimulation.	ETA, ADA, IFX	79 RA	Alzabin et al. (90).
AS	**Flow cytometry** Increased Th2, Th17 relative to healthy controls. No changes with treatment. Increased memory CD4+ cells after treatment. No alterations in CD8+ cells.	IFX	13 AS	Szalay et al. (91)
AS	**Flow cytometry, ELISA** Patients vs. controls: increased Th17, decreased Treg After therapy: decreased Th17 and increased Treg only in responders.	ETA, ADA, IFX	222 RA	Xueyi et al. (92)
RA	**Flow cytometry** Increased Th1, Th2, Th17; decreased Treg relative to healthy controls. Increased Treg and Th1 after treatment (IFX, ET only). No changes in Th17.	ETA, ADA, IFX	10 HC 51 RA	Szalay et al. (93)
RA	**Flow cytometry, ELISA, microarrays** Increased IL-17+CD4+, IL-10+CD4+ and IL-17+IL-10+ T cells after therapy. Increased expression of Aiolos transcription factor.	ETA, ADA, IFX	HC 31 RA 61	Evans et al. (94)
RA AS PsA	**Flow cytometry, ELISPOT analysis of PBMC** Increased frequency of total IL-17+ cells and of CD4+IL-17+ cells 12 weeks after initiation of therapy in RA and AS patients	ETA, ADA	25 RA 15 AS 8 PsA	Hull et al. (95)
RA	**Flow cytometry** Increased frequency of IFNγ+Tbet+CD4+, and of IL-17+RORγt+CD4+ T cells in patients vs. controls; decreased frequencies after treatment. Only non-responders upregulate IL-17 production in stimulated cultures.	IFX	10 HC 55 RA	Talotta et al. (96)
RA	**Synovial thickening and vascularity assessed by ultrasonography** **Flow cytometry, ELISPOT analysis of PBMC** Increased frequency of circulating Th17 cells after treatment, correlated with decreased joint inflammation. Higher frequency of circulating Th17 cells at baseline is associated with poor anti-TNF response.	ETA, ADA	25 RA	Hull et al. (97).
**REGULATORY T CELLS**				
RA	**Flow cytometry, functional assays** Frequency of Treg increases with treatment in responding patients. Treg from active RA have deficient suppressive activity. Suppressive activity is restored by anti-TNF treatment.	IFX	27 RA	Ehrenstein et al. (98)
RA	**Flow cytometry, proliferation assays** IFX therapy induces CD4^+^CD25^hi^FoxP3^+^ Treg, which suppress through TGFβ and IL-10	IFX	20 HC 31 RA	Nadkarni et al. (99)
RA	**Flow cytometry** After therapy: decreased CD4+CD25+ effector cells; increased frequency of Foxp3+CD4+CD25+ Treg	ETA	33 RA	Huang et al. (100)
RA	**Flow cytometry, ELISA, Treg cell suppression assays** Treg increase in ADA-treated responders, not with ETA treatment. Treg from ADA responders are suppressive, but not Treg from non-responders or patients treated with ETA.	ADA, ETA	15 HC 50 RA	McGovern et al. (101)
RA	**Flow cytometry** Decreased Treg in untreated patients. Treg are increased in IFX-responsive patients compared to untreated and non-responders.	IFX	10 HC 55 RA	Talotta et al. (96)
RA	**Flow cytometry, ELISA, functional assays** Increased Treg with ADA treatment ***in vitro***, not with ETA. Decreased Th17 after treatment. ADA increases mTNF on monocytes, which stimulate Treg expansion.	ADA, ETA	8 HC 26 RA	Nguyen and Ehrenstein (102)
**B CELLS**				
RA	**Flow cytometry, immunohistochemistry** Anti-TNF treated patients have reduced frequency of memory B cells, and increased naïve and transitional B cells. Anti-TNF treatments alters the lymphoid architecture (decreased germinal centers).	ETA	22 HC 45 RA	Anolik et al. (103)
RA	**Flow cytometry, ELISA, PCR** RA vs. HC: lower frequency of pre-switch memory B cells, increasing post-switch memory B cells with disease duration. Anti-TNF treatment increases pre-switch memory B cells frequency. Enhanced expression on memory B cells of CXCR1, CXCR2, CCR2	IFX	40 HC 56 RA	Souto-Carneiro et al. (104)
RA	**Flow cytometry, ELISPOT** decreased influenza-specific serum antibody and memory B cell responses (plasmablasts) in RA patients treated with anti-TNF.	ETA, ADA, IFX	97 HC 164 RA	Kobie et al. (105)
SpA	**Flow cytometry** Patients with anti-TNF therapy have increased memory B cells and B cell activation, reduced naïve B cells and impaired response to vaccination. Increased unswitched memory cells with decreased somatic hypermutations suggest a defect in germinal centers. No defects in T cell subsets.	ETA, ADA, IFX	56 SpA	Salinas et al. (106)
RAPsA	**Flow cytometry, multiplex assay** NK and B cell *numbers* reduced in patients vs. controls, increased after anti-TNF therapy in responders. No differences in B cell *frequencies*, untreated RA vs. control or vs. treated.	ETA	45HC 82 RA 32 PsA	Conigliaro et al. (107)
RA	**Flow cytometry** RA vs. controls: no differences in B cell subsets. Increased memory B cells in active disease. Increased total B cells after anti-TNF treatment. Responders have higher frequency of memory B cells at baseline.	ETA, ADA, CER	31 HC 96 RA	Daien et al. (108)
JIA	**Flow cytometry, ELISA** Anti-TNF increases circulating Tfh cells, no effect on B cell subsets	ETA	28 JIA	Glaesener et al. (109)
AS	**Flow cytometry, ELISA** AS vs. control: decreased number of circulating follicular helper T cells with defective function, and decreased number of plasmablasts. These alterations are absent in patients treated with anti-TNF.	ETA, ADA, IFX	50 HC 50 AS	Bautista-Caro et al. (110)
JIA	**Flow cytometry, ELISPOT** Decreased transitional B cells in patients vs. controls. Conserved mature B cell compartment in untreated and treated patients. Lower response to certain vaccines.	ETA, ADA, IFX, GOL	31 HC 46 JIA 4 other PRD	Ingelman-Sundberg HM et al. (111)
AS	**Flow cytometry** No differences in transitional B cell numbers between AS and controls, but AS cells are defective in IL-10 secretion.		15 HC 15 AS	Chen et al. (112)
RA	**Flow cytometry** RA vs. control: decreased transitional B cells. Decreased Th17 cells. No changes in B cells during treatment.	ETA, GOL, CER	17 HD 31 RA	Salomon et al. (113)
RA	**Flow cytometry, ELISA** Anti-TNF therapy reduces activation (CD69) of B cells, and increases frequency of IL-10+B cells. Total frequency of B cells, and serum IL-10 unaffected by anti-TNF.	ADA, ETA	16 RA	Bankó et al. (114)
PsAPSO	**Flow cytometry** Memory and transitional B cells decreased in patients vs. controls. IL-10+B cells decreased in patients vs. controls, inversely correlated with Th17 and Th1 cells. Anti-TNF increases memory B cells in PSO.	Unspecified TNF inhibitors	23 HC 60 PsA 50 PSO	Mavropoulos et al. (115)
AS	**Flow cytometry, ELISA** Increased transitional B cells in AS vs. controls, reduced after treatment (6 patients).	IFX, GOL, ADA, CER	42 HC 42 AS	Bautista-Caro et al. (110)
**INNATE CELLS AND ANTIGEN PRESENTING CELLS**				
RA	**Flow cytometry** RA vs. controls: decreased pDC, increased TNFα+ DC. No changes with anti-TNF therapy. Decreased number of CD4+, CD8+, CD3+ cells, normalized after anti-TNF therapy.	ADA	10 HC 10 RA	Dombrecht et al. (116)
RA	**Flow cytometry, functional assays** Anti-TNF treatment: decreased activation of NK cells and IFNγ production. No effect on NK numbers, subsets.	ETA, ADA, IFX	39 RA	Nocturne et al. (117)
RA	**Flow cytometry** Vδ2 γδT cells are low in RA blood, but accumulate in joints, and produce high levels IFN-γ and IL-17. ETA restores circulating numbers of Vδ2 cells and decreases the expression of chemotactic receptors CCR5 and CXCR3.	ETA	21 HC 67 RA 21 OA	Mo et al. (118)
SpA	**Flow cytometry, single cell qPCR, ELISPOT** NKp44+ ILC3s are enriched in inflamed joints. Upon restimulation these cells produced IL-22 and CSF-2, no IL-17a		14 HC 26 SpA 11 RA	Blijdorp et al. (119)
RA	**Flow cytometry, ELISA** Increased frequency of CD14+CD16+ monocytes in active RA vs. controls. Decrease after DMARD therapy.			Kawanaka et al. (120)
RA	**Flow cytometry** Increased CD14+CD16+ monocytes in RA vs. controls. Increased frequency of CD4+, CD8+, B cells, granulocytes. After anti-TNF therapy: decreased CD16+ granulocytes. No changes in monocytes, T or B cells.	IFX	22 HC 63 RA	Coulthard et al. (121)
RA	**Flow cytometry** Expansion in young RA vs. controls of CD14+CD56+ monocyte with inflammatory properties. Reduction of this population after anti-TNF treatment.	ETA	86 HC 75 RA	Krasselt et al. (122)
RA	**Gene expression analysis arrays** Anti-IL-6 and anti-TNFα regulate different types of lincRNAs in CD14+ monocytes ***in vivo***.	ADA	5 RA	Müller et al. (123)
RA	**Flow cytometry, ELISA, functional studies** Increased TNFR1+ and decreased CD54 expression on monocytes are associated with a good therapeutic response. tmTNF crosslinking induced decoy receptors (sTNFR1, sIL-1R1, and sIL-1R2), correlated with response.	ETA	18 RA	Meusch et al. (124)
RA AS	**Flow cytometry, ELISA** After anti-TNF treatment: increased CD14+CD16+; decreased CD14+CD16- monocytes. Reduced expression of CXCR4+, CCR2+ on non-classical monocytes. Decreased serum SDF1 (CXCR4 ligand) after treatment.	IFX	5 RA, 5 AS	Aeberli et al. (125)
ASRA	**Flow cytometry** AS: increased M2 monocytes, negatively correlated with CRP. Anti-TNF therapy decreases M1 monocyte frequency.	ETA	100 HC 120 AS 50 RA	Zhao et al. (126)
**ALL POPULATIONS**				
RA	**Flow cytometry** After treatment, increased CD4+ memory cells, increased CD45RA+CD27+ CD8 memory cells, increased CD4+ IFNγ+ Th1 cells.	IFX	17 RA	Maurice et al. (127)
AS	**Flow cytometry, ELISA** Decreased TNFα+ and IFNγ+ CD4 and CD8 cells after anti-TNF therapy.	IFX	20 AS	Zou et al. (128)
AS	**Flow cytometry, ELISA** Increased TNFα+ and IFNγ+ CD4 and CD8 cells after anti-TNF therapy	ETA	20 AS	Zou et al. (129)
RA	**Flow cytometry, multiplex ELISA** After therapy: decreased Th1; decreased Th17 in non-responders. Decreased CD8+IFNγ+. Increased CD56+IFNγ+ NK cells.	ADA	18 RA	Aravena et al. (130)
RA	**Flow cytometry** RA vs. control: decreased CD4+CD25+, increased Th2 Anti-TNF vs. untreated: decreased naïve CD8+ and CD4+, memory CD8+. Increased Treg Responders vs. non-responders: decreased CD4+ and activated CD4+ frequency. Increased Th1 and Th17 in responders vs. untreated. CD4+CD69+ T-cell percentage has highest probability to discriminate responders/non-responders.	ETA, GOL, CER, ADA, IFX	30 HC 92 RA	Dulic et al. (131)

*RA, Rheumatoid arthritis; AS, Ankylosing Spondylitis; SpA, Spondyloarthritis; JIA, Juvenile Idiopathic Arthritis; PsA, Psoriatic Arthritis; PSO, Psoriasis; PRD, Pediatric Rheumatic Disease; HD, healthy donors; ETA, etanercept; ADA, adalimumab; IFX, infliximab; CER, certolizumab; GOL, golimumab*.

**Table 2:** Cytokines/chemokines and anti-TNF treatment.

**Pathology**	**Cytokine/chemokine**	**Treatment**	**Subjects**	**References**
RA	**ELISA, enzyme-amplified sensitivity immunoassays** After treatment, decreased IL_1Ra, IL-6, transient decrease IL-1β	IFX	73 RA	Charles et al. (132)
RA	**Immunohistochemistry, ELISA** After treatment, decreased IL-8, MCP-1 in synovial biopsies	IFX	10 RA	Taylor et al. (133)
RA	**ELISA, RT-PCR, immunohistochemistry** After treatment, decreased IL-1Ra, IL-1β IL-6, TNFR1, TNFR2 mRNA levels in whole blood	anti-TNFα monoclonal (D2E7)	120 RA	Barrera et al. (134)
RA	**ELISA of serum** Decreased IL-8, RANTES, MCP-1 after first injection of anti-TNF.	IFX	15 RA	Klimiuk et al. (135)
RA	**ELISA analysis of serum and of culture supernatants from synovial cells** Decreased IL-23 and MIP-3a in serum after treatment. No significant decrease of IL-17.	ETA	22 RA	Kageyama et al. (136)
RA	**ELISA** CCL18 is elevated in RA, and decreases after treatment	IFX	41 HC 61 RA	van Lieshout et al. (137)
RA	**Protein biochip array on serum** High serum levels of MCP-1 and EGF were associated with a response to ETA. Trend to decrease of EGF, MCP-1 and IL-6 in responders.	ETA	33 RA	Fabre et al. (138)
RA	**ELISA analysis of serum and of culture supernatants from synovial cells** Higher serum levels of CCL20 in untreated RA patients, relative to healthy controls. Decrease of CCL20 levels after anti-TNF treatment.	IFX, ETA	14 RA	Kawashiri et al. (139)
RA	**ELISA, RT-PCR** Reduction of serum CX3CL1 and its receptor, CX3CR1, on CD8+ T lymphocytes in responsive patients.	IFX	20 RA	Odai et al. (140)
RA	**ELISA on whole blood stimulated culture** Decreased IL-8 and IFNγ with IFX, decreased IL-6 with ETA.	ETA, ADA	6 HC 13 RA	Popa (141)
RA, AS	**ELISA on serum** After therapy: decrease of MIP-1a in AS, not in RA	ETA, ADA	13 HC 8 RA 6 AS	Akbulut et al. (142)
RA	**ELISA serum, flow cytometry, RT-PCR** Decreased IL-6, no difference for IL-17 after treatment. Th17 correlate with disease activity, decreased after treatment.	ADA	20 RA	Yue (143)
RA	**ELISA** Elevated serum levels of IL-6, IL-17, IL-21, IL-23 and TNFα in active RA patients vs. healthy controls.	ETA, ADA	12 HC 48 RA	Chen et al. (86)
PSO, IBD	**Flow Cytometry, RT-PCR in PBMC and CD4**± **T cells** After anti-TNF treatment, increased production of IL-17 and Th1, Th2 cytokines by PBMC, increased TCR-induced T cell activation and proliferation. Downregulation of cytokine gene expression in skin lesions.	IFX, ETA, ADA	26 HC 43 PSO 10 IBD	Bosé et al. (144)
RA	**ELISA, flow cytometry** After therapy: decreased IL-1β, TNF-α, IL-17, IL-6, and IL-23; increased TGFβ. No changes in IFNγ.	ETA	40 RA	Lina et al. (88)
AS	**Multiplex ELISA** Elevated serum levels of IL-6, IL-12, IL-17A, IFNγ,TNFα, and IL-8 in patients vs. healthy controls. Decreased after anti-TNF treatment.	IFX, ETA	8 HC 16 AS	Limon-Camacho et al. (89)
AS	**ELISA on serum** Patients vs. controls: increased IL-6, IL-17, IL-23. Trend toward increased IL-12, TGFβ. No differences in serum cytokine levels between patients treated with anti-TNF or conventional therapy.	IFX, ETA, ADA	38 HC 127 AS	Taylan et al. (145)
RA	**ELISA on supernatant of stimulated whole blood cultures** RA vs. controls: decreased secretion of TNFα, IL-1β, IL-6. Il-1β is higher in responders. Secretion of TNFα, IL-1β, IL-6 increases after treatment.	IFX, ETA, ADA	12 HC 41 RA	Kayakabe et al. (146)
AS	**Flow cytometry, ELISA on serum** Patients vs. controls: increased TNFα, IL-6, IL-17, IL-23 After therapy: decreased TNFα IL-6, IL-17, IL-23 in responders only.	IFX, ETA, ADA	222 AS	Xueyi et al. (92)
RA	**ELISA** Decrease IL-34 after anti-TNF treatment.	IFX, ETA	55 HC 125 RA 40 OA	Tian et al. (147)
RA	**ELISA on plasma** Plasma CXCL13 decreases after treatment.	ADA	76 RA	Greisen et al. (148)
RA, AS, OA	**ELISA** Serum IL-34 is elevated in RA and AS vs. controls. Higher serum levels associated with progression.		59 HC 100 RA 19 OA	Chang et al. (149)
AS	**ELISA** Reduced serum E-selectin levels after therapy No significant changes in MCP-1 levels.	IFX	30 AS	Genre et al. (150)
AS	**Multiplex ELISA** Active AS had higher IL-23 and PGE2 plasma levels compared with control-AS and healthy controls. After 24 months anti-TNF PGE2, IL-23 remain elevated. IL-17: no differences AS vs. healthy controls. After treatment, no changes in average IL-17 of all patients. But responders have increased IL-17 than non-responders.	IFX, ETA, ADA	47 HC 86 AS	Milanez et al. (151)
RA	**ELISA on serum** CXCL10, CXCL13, and CCL20 levels are decreased after TNF inhibitor therapy.	ADA, ETA	29 RA	Han et al. (152)
JIA	**Electrochemiluminescent 4-plex and single-plex assays** After treatment increased serum IL-10 and TNFα, despite clinical improvement. IL-17 levels remain higher in ETA-treated patients, compared to ADA.	ETAADA	16 HC 47 JIA	Walters et al. (153)
RA	**Functional reporter cell assay on serum** Decrease in type I IFN activity after treatment Increased IFN-β/α activity ratio at baseline associated with lack of response to anti-TNF.	ETA, ADA, CER, GOL, IFX	124 RA	Wampler Muskardin et al. (154)
RA	**Flow cytometry, multiplex ELISA on stimulated T, B cells, monocytes** T cells from non-responders produce more TNFα and GM-CSF than responders before treatment Responders have higher plasma levels of GM-CSF before treatment (positive predictive value of 87.5%).	ETA, ADA, CER, GOL	97 RA	Bystrom et al. (155)
SpA	**Flow cytometry, cytOFF, RNA-seq on peripheral blood and synovial cells** Increased GM-CSF+ cells in SpA, compared to controls.		HC 17 SpA 38 RA 14	Al Mossawi et al. (156)
RA, PsA, OA	**Flow cytometry** Expanded GM-CSF+ B and T cells in untreated RA with active disease. Anti-TNF decreases the frequency of GM-CSF+ cells, not associated with response. Increased GM-CSF+ B and T cells in OA and PsA relative to healthy controls.	ETA, ADA, CER, GOL	16 HC 40 RA 10 PsA 15 OA	Makris et al. (157)

However, the effects of TNF inhibition on immune cell populations are not always consistent in different studies, and may vary depending on the disease setting, the treatment (often studies group patients treated with different inhibitors) and whether the patients respond to therapy.

### Anti-TNF and CD4^+^ T Cell Subsets

The ability of the immune system to mount efficient responses against an array of pathogens depends on the differentiation of naïve CD4^+^ T cells into functionally distinct T helper (Th) subsets, characterized by the secretion of specific “cytokine signatures.” Th1 cells secrete IFNγ and are important for host defense against intracellular pathogens, while Th2 cells produce IL-4, IL-5, IL-13, and are involved in the protection against parasitic infections. The more recently identified Th17 cells secrete IL-17, IL-21, IL-22, and IL-26 in humans, and contribute to immune responses against extracellular bacteria and fungi. Both Th1 and Th17 responses have been associated with autoimmune disease (162), and the interaction between Th1 and Th17-secreted cytokines may drive disease phenotypes. As a demonstration of the complexity of the cytokine network activated in inflammation, IFNγ was shown to inhibit Th17 differentiation (163, 164) and to affect susceptibility to IL-17-induced experimental arthritis in mouse models (165, 166). In humans, IFNγ production has been found to increase after TNF inhibition (85, 127, 167, 168), however both increased (85, 93, 127) and decreased (88, 89, 96) Th1 frequencies have been reported after anti-TNF therapy. The underlying causes for such discrepancies are not clear, and better patient stratification may allow to provide a better understanding of the mechanism of function of anti-TNF agents. As an example, Th1 cells were significantly increased after adalimumab treatment only in patients in remission, compared to patients with active RA (85, 93, 127). This effect was not visible when the global treated population was compared to the untreated one. Individual drugs may also have specific mechanisms of action: two studies conducted by the same laboratory have found both increased (128) and decreased (128) Th1 cells in the blood of AS patients treated with etanercept or infliximab, respectively. The authors suggested that this differential effect may be linked to the different efficacy of these compounds in the treatment of Crohn's disease, and in the distinct rates of tuberculosis infections following these treatments.

Similar contrasting data have been observed for Th17 cells, with the only consensus of increased levels of Th17 (and Th1) frequencies in patients vs. healthy controls (see Tables 1, 2). High baseline frequency of Th17 cells may be associated with poor response to anti-TNF in RA (86, 90, 97), suggesting that disease in these patients could be driven by a different cytokine network.

The increase of Th17 cells after TNF-blockade has also been correlated with lack of response to treatment in AS (92) or RA (86), although increased Th17 and Th1 have also been found in responders to adalimumab in RA (85). An expansion of Th17 cells is also present in animal models of chronic inflammatory diseases (158). In a model of collagen-induced arthritis, TNF-blockade caused increased expression of the cytokine p40 subunit shared by IL-12 and IL-23, and these cytokines, in turn, induced the expansion of Th1 and Th17 cells, respectively (169). Albazin et al. demonstrated a similar increase in IL-12/IL-23p40 in stimulated blood and lymphocytes from RA patients with poor responses to anti-TNF treatment, suggesting that a similar mechanism could be active in human disease (90).

In several studies, the decrease in Th subsets was mirrored by an increase in regulatory T cells (Treg), indicating that the balance between effector T cells and Treg is important for the re-establishment of immune homeostasis. Altered Treg functions have been described in IMID, and Treg frequency is often decreased in patients, compared to healthy donors. These defects can be reversed by anti-TNF therapy, depending on the agent used (11, 102, 158) (Table 1). Adalimumab was found to restore Treg function in RA patients by increasing mTNF expression on the surface of monocytes. The resulting interaction with TNFR2 on Treg promoted their expansion (102).

### Anti-TNF and Cytokines/Chemokines

Anti-TNF treatment downregulates the production of a large range of inflammatory cytokines/chemokine, including IL-6, IL-1β, IL-8, RANTES, and MCP-1 (Table 2).

More recently, granulocyte macrophage-colony stimulating factor (GM-CSF), a myelopoietic cytokine that induces myeloid cells activation and differentiation, has emerged as a potential target in the treatment of rheumatic diseases (170, 171).

GM-CSF gene expression tends to occur locally in inflamed tissues and can be induced by inflammatory cytokines such as IL-6, IL-1β, TNF-α in many different cell types, both immune (T cells, monocytes) and not (fibroblasts, chondrocytes and endothelial cells) (172). GM-CSF receptors are found mostly on the surface of myeloid cells but are also expressed by non-hematopoietic cells, including fibroblasts (172). GM-CSF may affect adaptive immune responses indirectly, by supporting differentiation and function of antigen-presenting cell, and regulating Th cell development (112, 173).

Overexpression of GM-CSF and the GM-CSF receptor has been found in synovial joints of RA patients (173, 174) and in SpA (156). In RA joints, GM-CSF is mainly produced by IFN-γ^+^CD4^+^ T cells (175). In SpA blood and synovial fluid, CD4^+^ T cells were the main source of GM-CSF, which they produced alone or in combination with IL-17. GM-CSF was also produced by CD8^+^, NK, and innate lymphoid cells (156). The frequency of circulating GM-CSF^+^ cells was decreased in RA patients after anti-TNF treatment (157). GM-CSF is currently being investigated as a target for treatment of RA patients with inadequate responses to DMARDs (176).

It should be noted that the immune balance observed in peripheral blood may not reflect what occurs in the inflamed tissues, where pathogenic immune cells accumulate. One of the mechanisms of action through which TNF inhibitors decrease joint inflammation is by inhibiting immune cell trafficking (177, 178), by regulating the expression of adhesion and chemotactic molecules, and their receptors (127, 179). Chemokines (such as IL-8 and MCP-1) have an important role in recruiting immune cells to the synovia. Th17 cells express the chemokine receptor CCR6, which is important for their recruitment to the inflamed tissues (180). RA patients responding to adalimumab had significantly lower CCR6 expression than patients with active disease (85). These data are consistent with the decreased serum expression of the CCR6 ligand, CCL20, in RA patients undergoing anti-TNF therapy (139), and suggest that TNF-blockade may induce rerouting of Th17 cells from inflamed tissues to peripheral blood. Another example of TNF action on trafficking is the reduction of CX3CL1, produced by the endothelium, and its receptor CX3CR1 on CD8^+^ T cells in RA patients under infliximab, which may result in reduced T cell recruitment to the synovia (140). Additional chemo attractants that are decreased by anti-TNF therapy are CCL18, a product of dendritic cells (137), CXCL10, a ligand for CXCR3 on Th1 lymphocytes and monocytes, and CXCL13, a chemokine that attracts B lymphocytes and follicular T helper cells. In RA patients CXCL10 may also be important for chemotaxis of B cells toward inflamed tissues, as suggested by their increased expression of the receptor CXCR3 compared to healthy controls, and their ability to migrate toward CXCL10 in a chemotaxis assay *in vitro* (181). Consistently, CXCL10, as well as CXCL13 were found elevated in RA patients, compared to healthy or inflammatory controls, and in particular CXCL10 levels correlated with disease severity (182, 183). Notably, CXCL10 and CXCL13 levels were decreased specifically in patients responding to anti-TNF treatment, supporting the notion that disruption of lymphocyte recruitment is one of the keys of success for anti-TNF therapy (137).

### Anti-TNF and Other T Cell Subsets

The analysis of the inflamed joints has also revealed the importance of additional lymphocytic populations in the pathogenesis of rheumatic inflammation. γδ T cells carrying the Vδ2 receptor chain have been shown to accumulate in inflamed synovia from RA patients, and to produce high levels of IL-17 and IFNγ. Interestingly, synovial enrichment of Vδ2 T cells went in parallel with depleted numbers of these cells in the periphery, which were restored by anti-TNF treatment. TNF-α blockade strongly downregulates expression of CCR5 and CXCR3 on Vδ2 T cell, interfering with trafficking of these cells to peripheral tissues (184). Although few studies have analyzed innate immune cell populations, these data suggest that innate cells may also be important in the pathogenesis of chronic rheumatic inflammation.

### B Cells

B cells play an important role in IMIDs, by producing antibodies and a range of cytokines with pro- (TNF, IL-6, IL-17) or anti-inflammatory (IL-10) functions (185).

B cells exit the bone marrow as “transitional” cells that give rise to mature/naïve B cells, memory (CD27+) B cells and antibody-secreting plasmacells. In secondary lymphoid tissue follicles B cells can be activated to class-switch the B Cell Receptor from IgM/IgD to IgG, IgA, or IgE (186).

Several studies have reported an alteration in the number of B cells and the distribution of B cell subsets in rheumatic diseases (Table 1). Alterations have been reported in the frequency of various subsets of memory B cells, or plasmablasts (104, 108, 187, 188). A decrease in the immature transitional subsets has been reported in RA, PsA, and JIA patients (111, 115), but not in AS patients (110). Mauri and colleagues demonstrated that the transitional subset identified by the CD19^+^CD24^high^CD38^high^ markers contains the largest fraction of IL-10-producing B cells upon CD40 ligation in human peripheral blood (189). These cells have been called “regulatory B cells” (Breg), and can have an anti-inflammatory role, by inhibiting Th1 and Th17 responses, and inducing the expansion of Type 1 regulatory T cells (Tr1) (190).

As for T cells, many discrepancies have been observed in the various studies addressing the phenotype of B cells in IMID (Table 1). These could be explained by many factors, including the different markers used to characterized B cell populations, the cohort size, and patients' characteristics, including type of pathology, disease duration, and treatment. Daien et al. demonstrated that patients under steroid treatment have higher frequencies of memory B cells, in particular post-switch memory B cells, and fewer naïve B cells, compared to patients not treated with steroids. Age and sex were also important confounders: gender significantly influenced the composition of CD27^−^IgD^+^ naïve, CD27^+^IgD^−^ post-switch memory, and CD27^−^IgD^−^ B cells, while age was negatively correlated with CD27^+^IgD^+^ pre-switch memory B cells (108). These authors found that few of the differences in B-cell composition between RA patients and controls were confirmed after adjusting for age, gender, and steroid dose.

The reports in the literature on the effects of anti-TNF therapy on B cells are also controversial. In this case, the patients' different response to therapy, and the possible redistribution of cell subsets between inflamed tissue and circulation may also affect the balance of cell subsets.

In some, but not all, cases, anti-TNF therapy caused an increase in total B cells (107), or in memory B cells (106, 115). In the blood, however, this increase can coexist with profound defects in B cell function, as demonstrated by the defective antibody response to vaccination (105, 106, 111, 191, 192). Weak responses are mainly reported for T-cell dependent vaccines, suggesting an indirect effect of TNF blockade on antibody production. Consistently, profound alterations of germinal center structure have been observed in etanercept-treated RA patients (103), which may be explained by the ability of the TNFR2-Ig decoy to neutralize both TNFα and Lymphotoxin α. This could result in reduced isotype switching and hypersomatic mutations, leading to impaired B cell maturation (106).

### Anti-TNF and Ectopic Lymphoid Structures (ELS)

Lymphoid infiltrates that form in peripheral organs in conditions of chronic inflammation may assume organized features of ELS, characterized by the segregation of T and B cell areas, the presence of high endothelial venules and of a network of follicular dendritic cells [reviewed in (193, 194)]. ELS formation is driven by a number of cytokines, such as member of the TNF superfamily (TNF-α and LT-α) (195–197) and chemokines, including CXCL13, CCL19, and CCL21, which may be found enriched in the inflamed synovia of RA and SpA patients (198–200). These structures are reminiscent of germinal centers and have been proposed to promote tissue-specific B cell responses, by facilitating class-switch and affinity maturation of locally produced antibodies in the inflamed synovia of RA patients (201). However, whether ELS are active initiator of disease or a consequence of the ongoing chronic inflammation is still debated. In particular, it has been observed that synovial lymphoid neogenesis often lacks some of the features of germinal centers, possibly due to the lack of expression of essential chemokines, such as CCL21 (199).

ELS are particularly prominent in severe forms of RA, Sjogren disease and systemic lupus erythematosus, but have also been described in patients with “sero-negative” inflammation, such as osteoarthritis, crystal-induced arthritis (199), and diseases of the SpA spectrum. Follicular lymphoid-like structures were observed in 2 out of 7 surgical hip specimens from patients with advanced ankylosing spondylitis (202), and in 13 of 27 synovial specimens from PsA patients with no correlation with disease severity or the extent of joint involvement. However, a regression of ELS was observed in PsA or RA patients successfully treated with etanercept or anti-TNFα antibodies, suggestive of a role for TNF-induced inflammation in the generation of these structures (198). In RA patients, in particular, the presence of synovial ELS was also shown to be an independent negative predictor of response to anti-TNF agents (198).

## Prediction of Immune Responses to Anti-TNF Therapy

### Baseline Patient Clinical Characteristics as Predictors of Therapeutic Response to Anti-TNF Therapy

One of the major challenges in SpA care remains the development of better clinical or biological markers able to aid in disease diagnosis, describe disease activity, and predict structural damage, and therapeutic responses to biological treatments.

In this review we will limit our discussion to predictors of clinical outcome to anti-TNF therapy (see Table 3). Two scoring systems are used to assess disease activity in SpA; the Bath Ankylosing Spondylitis Disease Activity Index (BASDAI) (220) and the Ankylosing Spondylitis Disease Activity Score (ASDAS) (221). The BASDAI is based exclusively on patient reporting. It does not include laboratory measurements of inflammation, such as CRP level, but is widely used in clinical practice. In contrast, the ASDAS is a combination of patient reporting and measurements of inflammation [C-reactive protein (CRP) or erythrocyte sedimentation rate (ESR)]. Of note, ranking of patients according to the disease activity determined with the BASDAI or the ASDAS is not identical and it has been suggested more recently that the BASDAI should be replaced with the ASDAS (222).

**Table 3:** Biomarkers predicting therapeutic responses to TNF-blockers.

**Pathology**	**Biomarker associated with positive clinical outcome to anti-TNF therapy**	**Target**	**Treatment agent**	**References**
SpA	Young age, short disease duration, high CRP, high ESR, low BASFI	Serum and clinical characteristics	Infliximab Etanercept	Rudwaleit et al. (203)
SpA	Young age, male gender, presence of peripheral arthritis, high patients' global assessment of disease activity, high CRP, high ESR	Serum and clinical characteristics	Infliximab Adalimumab Etanercept	Arends et al. (204)
SpA	Combination of CRP and SAA	Serum	Infliximab Etanercept	de Vries et al. (205)
SpA	Combination of age, HLA-B27 genotype, CRP level, functional status, presence of enthesitis and choice of therapy at baseline	Serum and clinical characteristics	Infliximab Golimumab	Vastesaeger et al. (206)
SpA	Male gender, low body mass index	Clinical characteristics	Infliximab Adalimumab Etanercept	Gremese et al. (207)
SpA	Non-smokers	Clinical characteristics	Infliximab Adalimumab Golimumab Etanercept	Glintborg et al. (208)
SpA	High CRP, IL-6, CTX-II and MMP-3 and low YLK-40	Serum and clinical characteristics	Infliximab Adalimumab Etanercept	Pedersen et al. (209)
SpA	High calprotectin and hs-CRP, but not MMP-3	Serum	Infliximab Etanercept	Turina et al. (210)
RA	High calprotectin	Serum	Infliximab Adalimumab Rituximab	Choi et al. (211)
RA	CXCL10 and CXCL13	Serum	Adalimumab Etanercept	Han et al. (152)
RA	Increased expression of IFN-response genes after therapy associates with poor clinical response	RNA from peripheral blood	Infliximab	van Baarsen et al. (212)
RA	Failure to decrease expression of inflammatory genes *(IL1B, NFKBIA, IL8, CCL4*) after therapy associates with poor clinical response	PBMCs	Etanercept	Koczan et al. (213)
RA	ITGAX expression	Blood monocytes	Etanercept	Stuhlmüller et al. (214)
IBD	Frequency of baseline plasma cells and macrophages increased in non-responders. TREM-1 expression in blood significantly higher in responders.	Colon biopsies and blood	Infliximab	Gaujoux et al. (215)
IBD	Increased baseline expression of oncostatin-M in non-responders	Colon biopsies	Infliximab	West et al. (216)
IBD	Cells expressing mTNF in the intestinal mucosa	*In vivo* imaging of the intestinal mucosa	Adalimumab	Atreya et al. (217)
SpA	Increased proportion of baseline Burkholderiales	Gut microbiota	Infliximab Adalimumab Etanercept	Bazin et al. (218)
RA	Increased histamine, glutamine, xanthurenic acid and ethanolamine in responders	Urine	Infliximab Etanercept	Kapoor et al. (219)

CRP or ESR are commonly used in clinical practice to help diagnose SpA, evaluate disease activity and predict therapeutic outcome to TNF inhibitors. However, both have low sensitivity and specificity (223). CRP levels are also increased by aging, anemia, infections and presence of immunoglobulins (224, 225). Furthermore, elevated CRP or ESR are only present in about 40–50% of patients with SpA (203) and a normal CRP or ESR does not exclude a diagnosis of SpA. In fact, normal levels of these markers are seen in some SpA patients with active disease.

Rudwaleit and colleagues reported the first systematic analysis of the parameters correlated with clinical responses to anti-TNF therapy in active ankylosing spondylitis patients in 2004. Analysis of clinical parameters in 99 AS patients treated with infliximab or etanercept revealed that the likelihood of achieving good responses to TNF blockers was significantly associated with young age and shorter disease duration, with increased markers of acute phase and higher disease activity (203).

A study performed on 220 SpA patients indicated that the highest response rate during anti-TNF therapy was achieved in those with elevated CRP or ESR levels at baseline, younger age, male gender, presence of peripheral arthritis and higher patient's assessment of disease activity (204). In a large prospective cohort of AS patients, De Vries and colleagues confirmed the predictive value of inflammatory markers, such as CRP and serum amyloid A (SAA), to monitor the efficacy of anti-TNF treatment, and to select the patients more likely to respond to anti-TNF therapy. The study was performed on 155 SpA patients (117 treated with etanercept, 38 with infliximab) and clearly demonstrated that all the markers of inflammation tested, and the BASDAI score were significantly decreased after anti-TNF therapy. However, only elevated baseline levels of CRP and SAA were shown to correlate to positive therapeutic outcome and to be a valuable instrument for prediction. A combination of elevated baseline levels of CRP and SAA resulted in the best prediction of clinical response (81%), which was not improved by the inclusion of baseline ESR levels (205). The same study also reported that high sensitivity CRP (hs-CRP) did not provide advantage over standard CRP measurement.

More recently, a report from Vastesaeger defined an algorithm in which the combination of HLA-B27 genotype, age, functional status, CRP level, the presence of enthesitis, and choice of therapy at baseline enabled good prediction of anti-TNF outcome in two large cohorts of SpA patients. Interestingly, the authors demonstrated that although each factor can be an independent predictor, the combination of the six selected variables outperformed the single predictive value (226). This study also suggested that single predictors may not be strong enough for decision-making in the individual patient.

Additional baseline characteristics that have been correlated to therapeutic outcome in SpA patients are body weight and smoking. A report confirmed the lower probability to respond to TNF inhibitors in female SpA patients, and found an association with high body mass index (BMI) and poor response to therapy, in particular for infliximab treated patients (207). In a large observational cohort of SpA patients established in Denmark, in which more than half of the patients were current or previous smokers, the authors reported that current and previous smokers had significantly poorer anti-TNF treatment responses as compared to non-smokers, and suggested that the negative impact of smoking may be linked to increased systemic inflammation, accelerated radiographic progression, decreased functional activity and reduced lung capacity (208). This study confirmed a previous report in RA patients, in which a lower rate of response to anti-TNF therapy among current smokers was observed, particularly in patients receiving infliximab (227).

The clinical characteristics described so far may help clinicians to guide treatment decisions in daily practice, however none of them has strong specificity.

### Predictive Markers in Blood and Tissues

Different strategies have been proposed in the past 15 years with the aim to identify markers predicting clinical responsiveness to anti-TNF therapy. However, results reported in the literature have not always been validated in subsequent studies. Many different strategies have been pursued to identify biomarkers, such as measuring soluble molecules in serum or applying a variety of molecular and genomic techniques. More recently, alternative approaches including molecular imaging with fluorescent antibodies, analysis composition of the gut microbiota and the hunt for biomarkers in tissues have been proposed. Since there are only limited numbers of studies performed in SpA patients, we will extend our discussion to other chronic inflammatory diseases, the results of which might also be relevant for SpA.

A report from Pedersen and colleagues investigated circulating biomarkers of inflammation including CRP, IL-6 and YKL-40, vascular endothelial growth factor (VEGF, a marker of angiogenesis), C-terminal crosslinking telopeptide of type II collagen (CTX-II), matrix metalloproteinase 3 (MMP-3), total aggrecan, cartilage oligomeric matrix protein and bone turnover in axSpA patients undergoing anti-TNF treatment. Patients that reached a major improvement in ASDAS at week 22 had higher baseline CRP, IL-6, CTX-II, and MMP-3 and lower YLK-40 levels as compared to partial or non-responders. BASDAI responders had higher baseline CRP and VEGF, and lower YLK-40 as compared to BASDAI non-responders (209).

Higher MMP3 and other matrix metalloproteinases such as MMP8 and MMP9 levels were repeatedly shown to reflect disease activity and response to treatment in SpA patients but the results were not always concordant when comparing different studies. Arends and colleagues showed that although serum MMP-3 significantly decreased after etanercept treatment in SpA patients, baseline levels were not superior to the predictive accuracy of ESR or CRP (228). In line with this result, Turina et al. more recently reported that calprotectin and hs-CRP, but not MMP-3, were good biomarkers for treatment response in SpA patients, confirming these results in a replication cohort (210, 229). From this report and other studies it appeared that the most reliable marker correlating with clinical parameters in SpA identified so far is calprotectin, an heterodimeric protein complex consisting of S100A8 and S100A9 subunits (previously called myeloid-related protein 8 and 14, respectively), which bind calcium and zinc in the cytosol of monocytes and granulocytes (230, 231). Myeloid-related protein 8 and 14 are damage-associated molecular pattern molecules (DAMPs), and have been reported to be upregulated in several autoimmune disorders (232, 233). Produced by myeloid cells and neutrophils, calprotectin is secreted in the transmigration of these cells through the endothelium to the inflamed tissues and promotes inflammation by activating leucocytes and endothelial cells (234). As shown in animal models, it is also a key factor in the pathogenicity of autoreactive IL-17–producing CD8^+^ T cells (235).

Elevated serum levels of calprotectin were reported in axSpA and reactive arthritis patients compared to controls, and treatment with anti-TNF agents significantly decreased calprotectin and hs-CRP levels already 2 weeks after infliximab treatment in SpA patients, but not in the placebo group (229). In addition to calprotectin, several biomarkers (hs-CRP, IL-6, pentraxin-3, α-2-macroglobulin, MMP-3, and VEGF) were assessed at baseline for their ability to predict response to anti-TNF therapy. Importantly, serum levels of calprotectin were the most accurate and reliable marker of treatment response in axSpA and peripheral SpA, and outperformed hs-CRP in some analyses.

In RA patients, baseline serum levels of myeloid-related proteins 8 and 14 were significantly higher in responders compared to non-responders. The study was done in three prospective cohorts, one treated with adalimumab, one with infliximab and one with a different therapy, the anti-CD20 antibody rituximab. MRP 8/14 levels were consistently higher in responders to targeted treatment, independently of the mechanism of action of the biologics (211). Interestingly, the predictive accuracy of baseline MRP 8/14 levels in the three groups was higher as compared to other baseline patient characteristics such as CRP, ESR, and DAS28. The ROC curve AUC for baseline MRP 8/14 levels was 0.688 in the adalimumab-treated patients, 0.791 in the infliximab group and 0.984 in the rituximab group. Another important point of the study was that while in good and moderate responders there was a significant decrease of calprotectin levels 4- and 16-weeks after initiation of anti-TNF therapy, the levels of the protein remained unchanged in the non-responder group. This suggests that serial measurement of MRP 8/14 serum complexes may be useful for monitoring the early changes after biological treatment, and may predict the clinical response over time. The advantage of measuring MRP 4/18 serum levels, apart from the low cost and accessibility of the complex in the serum, is that the protein is relatively stable at room temperature, and, contrary to other cytokines, can be measured in serum without cold storage of samples.

Other markers correlated to anti-TNF therapy outcome were reported in a small cohort of RA patients, in which serum levels of CXCL10, CXCL13, and CCL20 were measured by ELISA. Baseline levels of CXCL10 and CXCL13, but not CCL20 were significantly higher in responders as compared to non-responders and both markers were reduced after therapy in the responder group, however the results were not validated in independent cohorts of patients (152).

Most treatment prediction studies have focused on markers measured in serum. Multiplex analysis of cytokines and chemokines in patients' serum samples often result in measurements close or below the lower limit of quantification of the secreted proteins, giving rise to conflicting results. These molecules are often secreted in low abundance in cells at the steady state and are therefore difficult to measure in robust and reproducible assays.

Few studies have been performed at the transcriptomic level, to identify genes associated with clinical outcome to anti-TNF therapy. Van Barseen and colleagues reported that poor clinical response to anti-TNF therapy was associated with an increased IFN gene expression signature one to 2 months post-therapy in peripheral blood of RA patients. Higher level of expression of IFN response gene set such as *OAS1, LGALS3BP, MX2, OAS2*, and *SERPING1* were found in the poor responder group of a validation set of patients, and this was most evident at the 2-months post therapy time point. Interestingly, the combination of the five genes into one IFN response gene set improved the prediction accuracy over the single genes (212). In another study done in RA patients, a failure to downregulate key inflammatory genes (*IL1B, NFKBIA, IL8, CCL4*) in PBMCs after TNF-α blockade was associated with poor long-term clinical outcome (213). Investigation of blood monocytes in RA patients revealed molecular differences between responders and non-responders, both before and after anti-TNF therapy. Of note, expression of the integrin alpha X encoding gene (*ITGAX*, encoding CD11c) was significantly higher in responders at baseline and was able to predict the therapeutic outcome with 100% sensitivity and 91.7% specificity. Clinical responders revealed an almost complete reset to normal levels of inflammatory monocyte markers whereas in non-responders the levels of those genes remained elevated (214).

It was recently shown that the majority of the genes in a signature for response to anti-TNF in IBD patients show higher expression in immune cell subsets, compared to other cells present in the biopsy tissues, suggesting that resident or infiltrating leucocyte populations represent a good target to investigate responses to anti-TNF therapy. However, a clear cell population has not yet been implicated in the response (215).

A combined statistical deconvolution and meta-analysis methodology of IBD patients naïve to anti-TNF therapy showed that the genes most involved in response to therapy are expressed in the myeloid, B and T-cell subsets. Gaujoux et al. demonstrated that baseline proportions of intestinal plasmacells and inflammatory macrophages were significantly increased in non-responders, and this difference was maintained after treatment initiation. Interestingly, a good response to anti-TNF was associated with a strong decrease in both cell populations. The predictive power of baseline abundance of macrophages and plasmacells associated with failure to anti-TNF therapy obtained by the computational deconvolution was validated in plasma samples from an independent cohort of IBD patients. At the transcriptomic level, the chemokine ligand 7 (CCL7) and the chemokine receptor 2 (CCR2) were upregulated in biopsies obtained from non-responders, whereas no significant difference for these two genes was observed in blood. Increased levels of genes in the CCL7-CCR2 pathway suggest that in non-responders there is an increased recruitment of inflammatory TNF-secreting macrophages to the inflamed tissuers, which contributes to increased plasmacell abundance ultimately impacting clinical outcome. Interestingly, one of the upstream regulators of the CCL7-CCR2 axis, TREM-1, the expression of which was measured in the blood of 22 CD patients prior to anti-TNF therapy, was significantly downregulated in non-responders, and showed a high prediction accuracy.

Still in the context of IBD, a recent publication from the Powrie lab has demonstrated that oncostatin M (OSM) levels are higher in inflamed intestinal tissue of IBD patients with deep mucosal ulcerations (severe disease). Of particular interest was the observation that OSM gene expression levels were substantially higher in colonic biopsies of IBD patients refractory to anti-TNF therapy. These findings identified OSM as a potential biomarker of disease activity and therapeutic response to anti-TNF therapy. In addition, OSM could be an interesting novel therapeutic target for patients not adequately responding to TNF-blockers (216). However, it should be noted that this work has been performed with biopsies, material which is not available for most patients affected by axial SpA.

A study by Neurath and colleagues has recently used *in vivo* molecular imaging of the intestinal mucosa of Crohns' disease patients to identify mTNF-expressing cells. After topical application of labeled adalimumab through a standard spray catheter onto the most inflamed region of the bowel during colonoscopy, *in vivo* imaging of the intestinal mucosa showed a specific fluorescence signal of mTNF^+^ cells. Interestingly, cells expressing mTNF were mainly lamina propria CD14^+^ macrophages and some CD4^+^ T lymphocytes. The authors correlated the number of immune cells expressing mTNF with clinical outcome to adalimumab, and demonstrated that responders had a significantly higher mean number of mTNF-expressing cells than patients refractory to the therapy. This study was based on molecular imaging with fluorescent antibodies, which constitutes a new approach for the identification of patients responding to therapy, both in chronic inflammatory and autoimmune disorders, and in cancer (217).

More recently, several studies have addressed the role of metabolism and gut microbiome in patients with chronic inflammatory diseases and some reports have identified associations between baseline metabolites or microbiota composition and clinical responses to anti-TNF treatment. A recent study performed on a small cohort of axSpA patients reported an increased proportion of baseline Burkholderiales in clinical responders to anti-TNF therapy, and a high proportion of Dialister sp. at month 3 post-therapy (218).

Moreover, the screening of baseline urine metabolic profiles in a cohort of RA and PsA patients undergoing anti-TNF therapy allowed the identification of several metabolites associated with positive therapeutic outcome (219). The authors used different bioinformatics strategies to select the metabolites that better discriminate between responders and non-responders, and found that three different methods of analysis identified histamine, glutamine, xanthurenic acid, and ethanolamine as increased in urine samples from good responders to anti-TNF therapy, while ethanolamine, p-hydroxyphenylpyruvic acid, and phosphocreatine were lower in patients with a good response.

These studies suggest that the molecular analysis of serum or tissues may be a promising approach to identify biomarkers of response. However, a robustly validated and easily applicable biomarker is not available yet, in particular for SpA.

### Genetic Predictors of Treatment Response to Anti-TNF Therapy

The success of GWAS in identifying new disease susceptibility loci and indicating cellular pathways involved in pathogenesis has sparked interest to use the same approach to identify genetic determinants associated with treatment responses. Most of these studies have been performed in RA and only limited data are currently available for SpA.

In order to achieve optimal clinical outcome, treatment of RA needs to be initiated as soon as possible in newly diagnosed patients (236). According to the EULAR recommendations (236), most RA patients are treated with a conventional synthetic disease-modifying antirheumatic drug (csDMARD), such as methotrexate as first-line therapy, although this treatment is effective only in ~50% of patients. Patients who do not adequately respond or tolerate this treatment are subsequently switched to biologic drugs, such as TNF inhibitors (TNFi). Approximately 30% of RA patients do not respond to TNFi and are then prescribed another biologic therapy. This “trial and error” approach may take a long time, during which the patient is not appropriately treated but exposed to side effects without clinical benefit. More importantly, this approach is not compatible with the notion that treatment of RA needs to be initiated early in the disease. Therefore, identifying the most effective treatment for each individual patient at diagnosis is of critical importance to improve patient care. As biologic therapies are expensive, there is also a strong economic incentive to prescribe the most appropriate treatment early on.

Implementing “precision medicine” in rheumatic diseases will require robust tools that can predict responsiveness of patients to a specific drug. Genetic variants associated with therapeutic responses would be an ideal biomarker because these assays can be performed at diagnosis, are very robust, fast and inexpensive. A substantial number of studies have therefore been performed to identify genetic variants associated with treatment responses in RA (237). Some of these collaborative efforts employed candidate gene approaches to test if RA susceptibility alleles were also associated with therapeutic responses to TNF-blockers. Cui et al. tested the association of 31 RA risk alleles with therapeutic responses to TNFi in 1,253 RA patients from an international collaborative consortium of 9 different RA cohorts. They found that only the RA susceptibility allele (rs10919563) at the *PTPRC* locus (encoding CD45) was also associated with response to TNFi, but none of the other RA-associated risk alleles had an effect on treatment responses (238). These findings were replicated in a cohort of 1,115 patients from the UK (239). A third study, however, involving a meta-analysis of 1,516 patients did not find significant associations of *PTPRC* with response to anti-TNF therapy (240). Only few GWAS have revealed loci associated with treatment responses to anti-TNF therapy at accepted levels of genome-wide significance (*p* < 5 × 10^−8^). Cui et al. have performed a GWAS meta-analysis on 2,706 RA patients. 733 of these patients were treated with the soluble TNFR-Fc fusion etanercept, while 1,071 or 894 patients were treated with the monoclonal anti-TNF antibodies adalimumab or infliximab, respectively. They identified in the etanercept group, but not in the two other treatment groups a SNP in the 3′-untranslated region of the *CD84* gene, which may disrupt a transcription factor binding site. The allele associated with better treatment response was associated with higher gene expression levels of *CD84* and explained 2.6% variance in response to treatment with etanercept (241). The reason why this specific SNP at *CD84* was found to be associated only with therapeutic responses to etanercept but not to infliximab or adalimumab, is currently not known.

An innovative approach to identify a validated genetic predictor of anti-TNF response in RA has been performed in the context of the RA responder DREAM challenge (http://www.synapse.org/RA_Challenge). Genotyping data from 2,706 RA patients treated with anti-TNF were given to challenge participants to develop predictive models of treatment responses to TNFi. This challenge ran for 8 months in 2014 and 73 research teams submitted computer code covering a wide range of state-of-the-art modeling methodologies. In the validation phase, these models were evaluated with 591 anti-TNF-treated RA patients from an independent cohort. Despite the remarkable assembly of expertise in this crowdsourcing approach, no significant genetic predictors of treatment responses could be identified. The authors concluded that genetic information does not significantly enhance prediction of therapeutic responses over standard clinical assessments and suggested to embark on other research strategies to identify biomarkers (242).

## From IL-23/IL-17 biology to novel treatments of SpA and related diseases

GWAS data, together with mouse models of autoimmune disease, demonstrated that CD4^+^ inflammatory Th17 cells, which produce IL-17, play a pivotal role in the initiation of inflammatory diseases (243–245). IL-23 is important for the expansion and the functional activity of the Th17 cell subset (246). More recent studies have pointed to an additional role of IL-17-producing innate immune cells, which express the IL-23 receptor (IL-23R) in inflammatory disease. In particular, innate lymphoid cells (ILCs) were shown to drive IL-23-dependent intestinal inflammation in mice (247), and were enriched in the intestine of patients affected by inflammatory bowel disease (IBD) (248). In addition, a subpopulation of γδ T cells that produce IL-17 contributes to experimental autoimmune encephalomyelitis (EAE) in mice (249). IL-23R-expressing γδ T cells are also enriched in the peripheral blood of SpA patients (250). A direct link between IL-23 and tissue inflammation has been established in a mouse model of SpA. Sherlock et al. demonstrated that IL-23 mediates enthesal inflammation, the hallmark of SpA, by acting on a small population of CD3^+^CD4^−^CD8^−^IL-23R^+^RORγt^+^ enthesal resident T cells (251). The implication of the IL-23/IL-17 axis is also supported by the finding that at least 6 of the non-MHC loci genetically linked with axSpA are associated with genes in this pathway (*RUNX3, IL23R, IL6R, IL1R2, IL12B, TYK2*) (252). Taken together, these data suggest that the inflammatory response in SpA may be the result of a complex interplay of different immune cell types and that the IL-23/IL-17 pathway is likely to play a key role in chronic inflammation. Understanding the cellular and molecular mechanisms that regulate this network of innate and adaptive immune responses is therefore of critical importance for the design of rational therapies.

To address this question, our lab and others have investigated the impact of genetic polymorphisms in genes of the IL-23 signaling pathway on the effector functions of CD4^+^ T cells from SpA patients (253–255). We have measured the expression levels of Th17 and Th1 cytokine genes and transcription factors in CD4^+^ T cells isolated from SpA patients, and we correlated them with the patients' genotype at loci genetically associated with SpA. We showed that SpA patients carrying risk-associated alleles of genes in the IL-23/IL-17 pathway expressed high levels of genes involved in the differentiation and function of Th17 and Th1 cells, whereas the presence of protective alleles was associated with low-level expression of these genes. In contrast, variation at loci genetically linked to SpA, but not associated with the IL-23 pathway (such as *ERAP1* and *ANTXR2*), did not correlate with expression of Th17 and Th1 genes, suggesting that these SNPs may contribute to SpA pathogenesis through distinct cellular mechanisms. These data showed that genetic variation at multiple loci within the IL-23/Th17 pathway, such as *IL23R, IL12B*, and *CCR6*, affects CD4^+^ effector functions in SpA patients. Of note, the effect of genetic variation on CD4^+^ T cell function could be detected in activated, but not in resting T cells, consistent with the context-dependent action of expression quantitative trait loci (eQTL) observed in several studies (256–258). We also showed that the combinatorial action of multiple SNPs at distinct loci, rather than a single genetic variant, determined the immune cell functions of SpA patients and we have established a hierarchy among the SNPs with respect to their effect on regulating the expression of effector molecules using multivariate analysis. These results demonstrate a link between disease-associated genetic variants and defined functions of immune cell populations involved in the pathogenesis of chronic inflammatory diseases.

A large number of clinical trials have been performed in the past years to evaluate if targeting IL-23 and IL-17 is beneficial for the treatment of SpA and related diseases (259).

As mentioned above, the only treatment options for SpA patients not adequately or not tolerating treatment with NSAIDs have been TNF-blockers. The rationale to test IL-17A blockade were the strong genetic association of loci linked to the IL-23/IL-17 axis and the expansion of circulating CD4^+^IL-17^+^ cells in AS (260, 261), including KIR3DL2-expressing T cells responding to cell-surface HLA-B27 homodimers (262) and IL-17-producing γδ T cells expressing the IL-23R (250). Baeten and colleagues performed a phase 2 study to determine the efficacy and safety of secukinumab, a monoclonal antibody blocking IL-17A, in patients with active AS. This proof-of-concept study showed that inhibiting IL-17A rapidly reduced clinical and biological signs of active AS when compared to placebo and was well tolerated (263). Two subsequent phase 3 trials confirmed that inhibition of IL-17A significantly reduced signs and symptoms of AS (264), and this treatment is now recommended for the treatment of axSpA not adequately responding to TNF-inhibitors (265). Phase 3 trials have also documented efficacy of anti-IL-17A therapy for the treatment of psoriasis and psoriatic arthritis (266–268).

In contrast, inhibition of IL-17A with secukinumab was not effective and higher rates of adverse events were noted compared with placebo in a proof-of-concept study of Crohn's disease (269). A subsequent phase 2 study evaluated safety and efficacy of brodalumab, a human monoclonal antibody targeting the IL-17RA that blocks the biologic activity of multiple IL-17 cytokines including IL-17A, IL-17F, and the IL-17A/F heterodimer, in patients with moderate-to-severe Crohn's disease. This study was terminated early because a disproportionate number of cases of worsening of Crohn's disease in the treatment groups compared with placebo was observed (270). The precise mechanisms for the unexpected failure of IL-17A inhibitors in Crohn's disease are currently not known. However, Lee and colleagues recently reported that the dominant function of IL-17A in a mouse model of colitis is to preserve the integrity of the intestinal barrier by inducing expression of the tight junction protein occluding during epithelial injury (271).

To start to define the mechanism of action of IL-17A inhibitors, van Mens and colleagues have analyzed the effects of IL-17A inhibition on the immunopathology of target lesions and systemic immune responses in peripheral SpA. They reported that clinical improvement in joint counts was associated with a histologic decrease in synovial sublining macrophages and neutrophils, as well as with decreased synovial expression of *IL17A* but not of *TNF* transcripts. Systemically, anti-IL-17A treatment decreased inflammatory markers such as CRP and ESR and MMP-3 production in whole-blood stimulation assays using SEB and zymosan as stimuli. However, with exception of IL-17A itself, the capacity of peripheral blood cells to produce a broad panel of cytokines and chemokines upon stimulation with microbial antigens was not affected. This mechanism-of-action study was conducted in 20 peripheral SpA patients and indicated that clinical improvement upon anti-IL-17A treatment was paralleled by immunomodulation of inflamed target tissues without compromising systemic immune responses (272).

The crucial role of IL-17A in the pathogenesis of SpA has been demonstrated in clinical trials of the anti-IL-17A antibody secukinumab, however the cellular source of this proinflammatory cytokine in this disease remained controversial. Group 3 innate lymphoid cells (ILC3s) have been identified in several tissues as potent producers of proinflamatory cytokines, including IL-17A and IL-22. In collaboration with the team of D. Baeten we have recently characterized the presence and composition of ILCs and investigated if these cells are an important source of IL-17A in the synovial tissue of patients with peripheral SpA. We analyzed matched synovial tissue (ST), synovial fluid, and peripheral blood from SpA patients with actively inflamed knee joints. We found that ILCs, and in particular NKp44^+^ ILC3s, were expanded in inflamed arthritic joints. Single-cell gene expression analysis demonstrated that ST ILCs were clearly distinguishable from ST T cells and from their peripheral blood counterparts. We detected expression of the Th17 signature transcripts *RORC, AHR* and *IL23R* in a large fraction of ST ILC3s. These cells were capable to induce IL-22 and CSF2 but not IL-17A expression in response to *in vitro* re-stimulation. This study demonstrated that ILC3s were absolutely and relatively enriched in the synovial joint of patients with SpA, however these cells are not a significant source of IL-17A in this pathology (119). Thus, further studies are needed to define the cellular sources of IL-17A in this disease.

With respect to IL-23, a phase 2 clinical study tested the safety and efficacy of a fully human monoclonal antibody (ustekinumab) targeting the p40 subunit shared by IL-12 and IL-23 in psoriasis (273). This study revealed a 75% improvement in the psoriasis area-and-severity index (PASI) at week 12 in up to 80% of patients and a 90% improvement in 50% of anti-IL-12p40-treated patients (273). These remarkable results were confirmed in two subsequent phase 3 studies (274, 275). Of note, blocking IL-12/IL-23 by ustekinumab was more effective for the treatment of psoriasis than treatment with the TNF-blocker etanercept (276) and has shifted the treatment paradigm for this disease affecting 2–3% of the population. Treatment with ustekinumab blocks the bioactivity of both IL-12 and IL-23. Subsequent studies with a monoclonal antibody targeting the p19 subunit of IL-23 (risankizumab; neutralizing selectively IL-23 bioactivity) demonstrated an even higher efficacy in the treatment of psoriasis than blocking both IL-12 and IL-23 (277). Although neither IL-17A inhibitors nor IL-23 blockers result in a cure of psoriasis, the impact of these new drugs on the quality of life of patients cannot be overstated. It is also important to note that in this case the clinical observations in patients confirmed previous results obtained in experimental mouse models. Mice with a deletion of the IL-23p19 subunit, but not mice with a deletion of the IL-12p35 subunit were protected from disease in several experimental models of autoimmunity, such as experimental autoimmune encephalomyelitis (EAE) (244, 245), collagen-induced arthritis (CIA) (278), and inflammatory bowel disease (IBD) (279, 280).

Blocking the activity of IL-12/23 and of IL-23 has also been tested in moderately to severely active Crohn's disease and patients receiving either of these monoclonal antibodies had significantly higher response rates than did those receiving placebo (281, 282). In contrast, a phase 2 study testing the IL-23 inhibitor risankizumab did not demonstrate any evidence of clinically meaningful improvements compared to placebo in patients with active ankylosing spondylitis, although a significant reduction of the inflammatory marker CRP was observed (283). These findings were unexpected for several reasons. First, genetic variants in *IL23R* have been associated with AS (284) and this finding had been replicated in several GWAS (285, 286). Second, treatment of AS with IL-17A inhibitors has proven to be effective (263, 264) and IL-17A was shown to be downstream of IL-23 (243). Finally, overexpression of IL-23 in a mouse model induced a SpA-like phenotype (251). The reasons underlying the failure of IL-23 blockade in SpA are currently unclear. It is possible that IL-23-independent of sources of IL-17A, such as mucosal-associated invariant T (MAIT) cells play important roles in SpA pathology (287), or that IL-23 is important at the initiation of the disease, but not in established disease, as has recently been demonstrated in HLA-B27 transgenic rats, an experimental model of SpA (288). Clearly, more work needs to be done to resolve this intriguing issue.

## Conclusions

The clinical relevance of a “personalized” approach in medicine is well-accepted. However, despite the well-known individual variability in the susceptibility to infections and inflammatory diseases, disease progression and response to therapy, medical practice and public health policies typically take a “one size fits all” approach to disease management. This is due to a lack of understanding of what determines the individual predisposition to disease and the mechanisms associated with the response to a specific therapy. Biological strategies that block specific immune mediators such as TNF, IL-6, IL-17A, IL-23, or JAKs are effective only in a subpopulation of patients and can be associated with serious side effects. To improve clinical outcome, tools that allow prediction of treatment responses are needed. In addition, a better understanding of the pathogenic mechanisms will permit a more efficient use of existing therapies, as well as the development of novel targeted therapies. In a context in which new treatments for chronic inflammatory diseases have recently been introduced in the clinics, we highlight the importance of establishing a path toward personalized medicine by defining immunological correlates associated with therapeutic responses to anti-TNF therapy in SpA and related diseases.

The limitations of developing reliable biomarkers that can be used in daily practice derive from several factors. Many of the studies reviewed here have been performed in different centers and in relatively small cohorts of patients, limiting the unbiased identification of robust and validated biomarkers. In this sense, increased collaboration between centers and “merging” of cohorts of patients affected by IMIDs to increase sample size and power may help to define more reliably define biomarkers. “Consortium science” has been key to the success of large GWA studies aimed at identifying risk alleles for various diseases and should be reinforced as a valuable strategy in translational research.

Due to limited patient numbers, several studies have grouped patients not homogeneous for disease status, treatment agent, response to therapy, and other patient characteristics. Most of these variables have significant effects on the measurable parameters of patient immune functions, and are important confounders for the analysis of the impact of TNF-blockade on the immune system. This may be the cause underlining the many controversial findings reported in the literature about the modifications in immune function imparted by TNFi. Accurate patient stratification may allow a better understanding of the molecular and functional consequences of anti-TNF therapy, and improve the chances to identify strong biomarkers of response.

With respect to axial SpA, an additional challenge is provided by the difficult access to inflamed axial tissues. Sequential sampling during treatment in large cohorts of patients is almost impossible to perform, except for SpA patients with a predominant peripheral inflammation. In this particular case, peripheral blood still remains the only easily accessible material to study pathophysiologic processes and mechanisms contributing to the response to therapies.

Finally, recent clinical trials testing the safety and efficacy of monoclonal antibodies targeting IL-17A and IL-23 in SpA, psoriasis, psoriatic arthritis and Crohn's disease have been very encouraging and have increased the therapeutic options for these diseases. At the same time, their introduction into the clinics has increased the urgency to implement personalized treatment strategies. A trial and error approach, as it is currently applied in most cases, is simply not compatible with optimal patient care and efficient use of resources. However, defining objective criteria to guide treatment decisions for each individual patient remains a major challenge. Furthermore, clinical studies testing blockade of IL-17A and IL-23 have not been without surprises. The unexpected failures of anti-IL-17A in Crohn's disease and of anti-IL-23 in AS are stark reminders of our limited understanding of the pathogenic mechanisms of these diseases and are a call to action to all of us in the biomedical research community.

## Author Contributions

All authors listed have made a substantial, direct and intellectual contribution to the work, and approved it for publication.

### Conflict of Interest Statement

The authors declare that the research was conducted in the absence of any commercial or financial relationships that could be construed as a potential conflict of interest.
